# Aquaporins Alteration Profiles Revealed Different Actions of Senna, Sennosides, and Sennoside A in Diarrhea-Rats

**DOI:** 10.3390/ijms19103210

**Published:** 2018-10-17

**Authors:** Yixin Cao, Ying He, Cong Wei, Jing Li, Lejing Qu, Huiqin Zhang, Ying Cheng, Boling Qiao

**Affiliations:** 1 Key Laboratory of Resource Biology and Modern Biotechnology in Western China, Ministry of Education, Northwest University, No. 229 TaiBai North Road, Xi’an 710069, China; yixcao@163.com (Y.C.); m15289471859@163.com (Y.H.); 18235431656@163.com (C.W.); lijing_0221@163.com (J.L.); qlj3697@163.com (L.Q.); zhq15619395612@163.com (H.Z.); 15829758592@163.com (Y.C.); 2 Shaanxi Traditional Chinese Medicine Innovation Engineering Technology Research Center, No. 229 Taibai North Road, Xi’an 710069, China

**Keywords:** senna, sennosides, sennoside A, toxicity on kidney and liver, aquaporins expression profiles

## Abstract

Senna and its main components sennosides are well-known effective laxative drugs and are used in the treatment of intestinal constipation in the world. Their potential side effects have attracted more attention in clinics but have little scientific justification. In this study, senna extract (SE), sennosides (SS), and sennoside A (SA) were prepared and used to generate diarrhea rats. The diarrhea rats were investigated with behaviors, clinical signs, organ index, pathological examination, and gene expression on multiple aquaporins (*Aqps*) including *Aqp1*, *Aqp2*, *Aqp3*, *Aqp4*, *Aqp5*, *Aqp6*, *Aqp7*, *Aqp8*, *Aqp9*, and *Aqp11*. Using qRT-PCR, the *Aqp* expression profiles were constructed for six organs including colon, kidney, liver, spleen, lung, and stomach. The *Aqp* alteration profiles were characterized and was performed with Principle Component Analysis (PCA). The SE treatments on the rats resulted in a significant body weight loss (*p* < 0.001), significant increases (*p* < 0.001) on the kidney index (27.72%) and liver index (42.55%), and distinguished changes with up-regulation on *Aqps* expressions in the kidneys and livers. The SS treatments showed prominent laxative actions and down regulation on *Aqps* expression in the colons. The study results indicated that the SE had more influence/toxicity on the kidneys and livers. The SS showed more powerful actions on the colons. We suggest that the caution should be particularly exercised in the patients with kidney and liver diseases when chronic using senna-based products.

## 1. Introduction 

Senna (*Cassia angustifolia* Vahl or *Cassis acutifolia* Delile), which is a well-known irritant laxative, is widely used in the treatment of intestinal constipation throughout the world [1,2]. Its main bioactive components, sennosides, belong to anthranoid glycosides and consist of a variety of anthraquinone derivatives including sennoside A, B, C, and D. Due to their natural origin and effectiveness, senna extracts and sennosides are thought to have low oral toxicity and are popular for long-term administration. However, the administration of senna extracts has been reported with some side effects such as abdominal cramping, electrolyte and fluid deficiencies, flatulence, nausea, bloating, vomiting, incontinence, and malabsorption, [3], which have little scientific justification. The main reason is that the laxative function of senna products are resulted from the metabolites produced by the anthranoid glycosides in intestines. For example, sennoside A could be metabolized into rhein, emodin, and aloe-emodin [4]. This process makes complex metabolites for senna products, which brings more challenges to study senna laxative function and toxicity. 

In the last decade, the study of structure, localization, and function of aquaporins (AQPs) has attracted more attention from researchers. AQPs are a larger family of integral membrane proteins that form pores to function as regulators of intracellular and intercellular water flow [5]. Thirteen known types of aquaporins (AQP0–AQP12) were identified in mammals and are classified into three main subfamilies in terms of their functional characteristics. AQPs (AQP0, AQP1, AQP2, AQP4, AQP5, AQP6, and AQP8) are water-specific channels. Aqua-glyceroporins (AQP3, AQP7, AQP9, and AQP10) are permeable to water and/or other small uncharged molecules (ammonia, urea, and, in particular, glycerol) while AQP11 and AQP12 are named with superaquaporins regarding their lower identities on structures with others (less than 15%) [6,7]. These AQPs are differentially co-expressed in cells and various organs including colon, kidney, liver, lung, stomach, and spleen, etc. They mediate the bidirectional water flow in the organs by altering the AQP density in a particular membrane. No single AQP isoform is exclusively expressed at any single site [8]. Thus, these AQP sub-types bring approaches together and play key roles on water and fluid homeostasis in the human body. The current available data have indicated that AQPs expression changes are associated with constipation and diarrhea [9].

Being a laxative drug, senna has been reported with the regulation functions on water transport in the colon through AQP3 [9,10]. However, these research studies were based upon a single AQP gene only. To date, at least 11 AQPs have been found to be present in the small and large intestines and AQP3 is also expressed in other organs [11]. In addition, the complex metabolite mixtures would have potentials to target multiple AQPs. Therefore, we presumed that the AQP expressions might be regulated by the different metabolite mixtures and, thus, differ depending on the product of senna.

In this study, we generated rat models with different diarrhea grades by using three senna products including the senna extract (SE), sennosides (SS), and single compound, sennoside A (SA). Aiming to look at the potential influence from the treatment with the senna products, we investigated the diarrhea-rats with *Aqps* expressions on six organs including colon, liver, kidney, spleen, stomach, and lung. Using real-time quantitative reverse transcription polymerase chain reaction (qRT-PCR), we constructed mRNA alteration profiles for each organ in ten *Aqp* subtypes including *Aqp1*, *Aqp2*, *Aqp3*, *Aqp4*, *Aqp5*, *Aqp6*, *Aqp7*, *Aqp8*, *Aqp9*, and *Aqp11*. The Principle Component Analysis (PCA) was performed on the *Aqps* expression profiles to characterize the actions for each treatment. The overall influence was evaluated based on a series of clinical signs, organ index, pathological examination, the *Aqp* alteration profiles, and PCA analysis.

## 2. Results

### 2.1. Diarrhea-Rats Generated by Using the Senna Extract (SE), Sennosides (SS), and Sennoside A (SA)

The chemical components in senna extracts have been extensively analyzed in many studies. Sennosides are thought to be laxative components consisting of a variety of anthraquinone derivatives including sennoside A, B, C, and D. Among these constituents, sennoside A is thought to be a main laxative component and is widely used as a chemical marker for quality control to senna products. Thus, to clarify their quality, we detected content of SA in the SE and SS by using the High Performance Liquid Chromatography (HPLC) method described in the Materials and Method section. HPLC analysis indicated that the content of SA was 1.20% (*w*/*w*) in the SE and 8.13% (*w*/*w*) in the SS, respectively (data available if required). Clearly, there was a higher content of SA in the SS than in the SE.

Using the SE, SS, and SA, we wanted to generate diarrhea-rats with a low and a high grade. The diarrhea grade was assessed based on the fecal stool consistency, which was set as normal (grade 0), loose stool (grade +), and watery diarrhea (grade ++). Table 1 shows the diarrhea rats with diarrhea grade along with the administration used. The administrations with the low dose of SE, SS, and SA resulted in diarrhea with loose stool (grade +) and with the high dose of SE and SS led to diarrhea with watery diarrhea (grade ++). 

**Figure 1:**
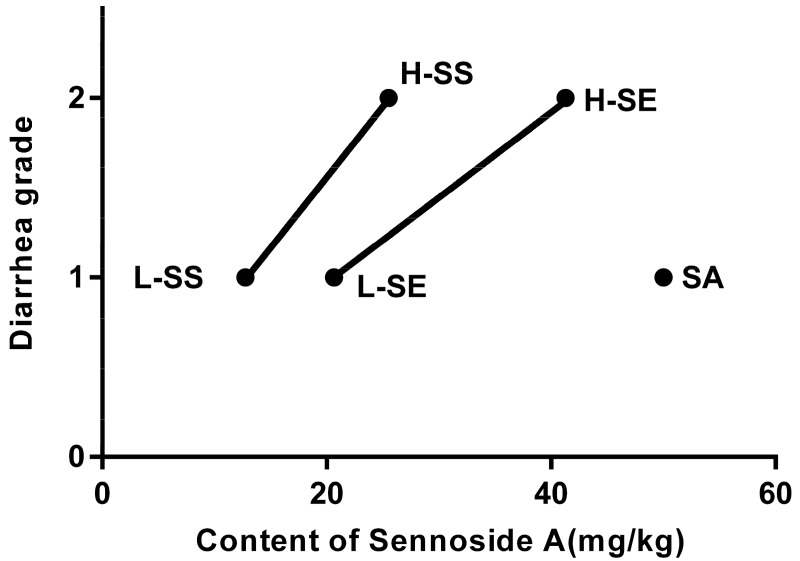
The diarrhea grade in the rats treated with a low dose of senna extract (L-SE), high dose of senna extract (H-SE), low dose of sennosides (L-SS), high dose of sennosides (H-SS), and sennoside A (SA) for over six days. The horizontal axis represents the content of sennoside A in each administration. The vertical axis depicts the diarrhea grade (1, 2 refers to loose stool and watery diarrhea, respectively).

Obviously, a smaller amount of the SS was required to generate the diarrhea model in rats. First, regarding the amount of dried powder, the low (L-SE) and high dose (H-SE) was 1725.00 and 3450.00 mg/kg, respectively. The low (L-SS) and high dose (H-SS) was 156.94 and 313.88 mg/kg, respectively. Second, based on the content of SA, the dose of the SE is two times more than that of the SS. Figure 1 shows the diarrhea grade along with their SA content. The SA content in the L-SE is 20.65 mg/kg and in the L-SS is 12.76 mg/kg. The H-SE value is 41.30 mg/kg and the H-SS value is 25.52 mg/kg. The single compound SA at 50.00 mg/kg achieved low diarrhea grade only.

### 2.2. The Body Weight Lost in the Diarrhea-Rats Induced by the SE But Not by the SS and SA

During and after administrations with the SE, SS, and SS, the rats were recorded with their body weight and clinical signs including behavior and diet. 

**Figure 2:**
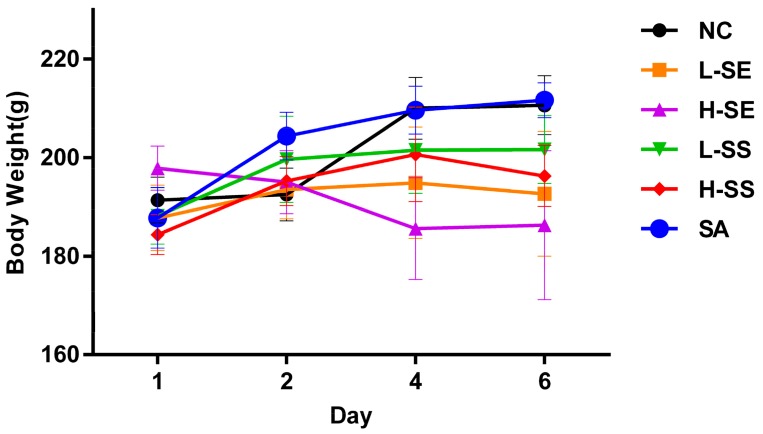
The transition of body weight (mean ± SD) in normal control (NC) rats and diarrhea rats induced by the low dose of senna extract (L-SE), high dose of senna extract (H-SE), low dose of sennosides (L-SS), high dose of sennosides (H-SS), and sennoside A (SA) for over six days. L-SE had a significant reduction on the mean body weight at day 4 (*p* < 0.01) and day 6 (*p* < 0.01). H-SE had a significant reduction on the mean body weight at day 4 (*p* < 0.001) and day 6 (*p* < 0.001).

The normal control group exhibited normal growth throughout the study while the decreases on body weight were found in the rats treated with the SE in both doses (L-SE and H-SE). The rats in the L-SE and H-SE groups had significant reductions on the mean body weight especially at day 4 (*p* = 0.006, *p* < 0.001) as well as at day 6 (*p* = 0.001, *p* < 0.001), respectively. There was a slight decrease in the growth of the rats treated with both doses of the SS without a statistical difference. No influence was found in the rats treated with the SA (Figure 2).

On the other hand, a series of clinical signs were shown in the diarrhea-rats indicating potential side-effects that resulted from the administrations. The rats treated with the SA and the low dose of SE and SS showed slight hair removal and diet reduction while, with the high dose of SE and SS, it showed severe hair removal, diet reduction, and even mental malaise.

### 2.3. Increased Organ Index in the Diarrhea-Rats Induced by the SE, SS, and SA

**Figure 3:**
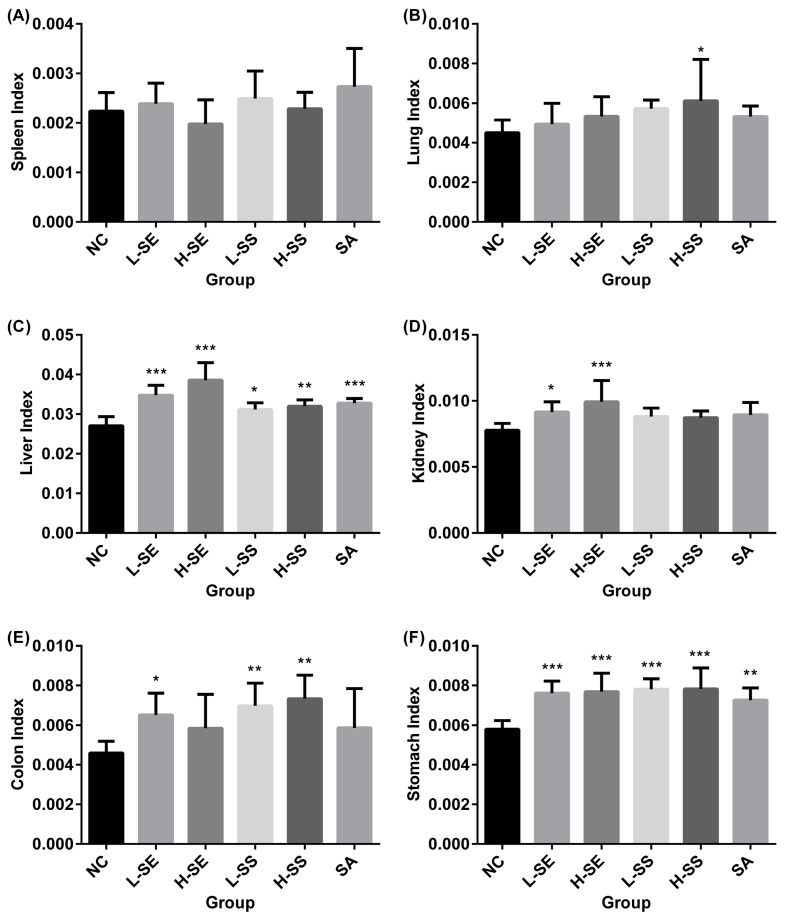
The organ index of normal control (NC) rats and diarrhea rats induced by the low dose of senna extract (L-SE), high dose of senna extract (H-SE), low dose of sennosides (L-SS), and high dose of sennosides (H-SS) and sennoside A (SA) for over 6 days. * represents a significant difference with the *p* < 0.05, ** represents significant difference with the *p* < 0.01, and *** represents significant difference with the *p* < 0.001. (**A**) The spleen index; (**B**) The lung index; (**C**) The liver index; (**D**) The kidney index; (**E**) The colon index; (**F**) The stomach index.

At day 6, the experimental rats were sacrificed and collected with six organs including colon, stomach, lung, kidney, lung, and liver. The tissues were weighted immediately, which was followed by further processing for other experiments. Figure 3 describes the organ index of the rats involved in the normal group and diarrhea groups.

Overall, there is no significant difference on the organ index with the spleens (Figure 3A) and the lungs (Figure 3B) between the normal group and all the diarrhea groups. However, others were found with increases in the diarrhea groups.

The significant increase was found with the liver index in all the diarrhea groups (Figure 3C). The SE treatments resulted in a dose-dependent manner with an increase at 28.48% in the L-SE (*p* < 0.001) and at 42.55% in the H-SE (*p* < 0.001). No big difference was observed in rats treated with the SS on both. The increase was 15.12% in the L-SS (*p* = 0.012) and 18.26% in the H-SS (*p* < 0.01). The treatment with the single compound SA caused an increase at 21.11% (*p* < 0.001).

The kidney index was significantly increased in the diarrhea rats induced by the SE used (Figure 3D). The increase was 17.78% with the L-SE treatment (*p* = 0.021) and 27.72% with the H-SE treatment (*p* < 0.001). No statistical significant difference was found in rats treated with the SA and the SS on both.

For the colon, all the treatments resulted in an increased colon index (Figure 3E). However, the statistical significance difference was shown in group L-SE and SS on both. The increase was 41.88% in the L-SE (*p* = 0.031), 51.82% in the L-SS (*p* = 0.005) and 59.62% in the H-SS (*p* = 0.001). The H-SE and SA had an influence with an increase around 27% without statistical significance. 

For the stomach, the significant increase with 30% was shown in all the diarrhea groups (*p* < 0.01). However, no dose-dependent manner was found in all the treatments (Figure 3F). 

### 2.4. Histopathological Changes in Colons Collected from the Diarrhea-Rats Induced by the SE, SS, and SA

**Figure 4:**
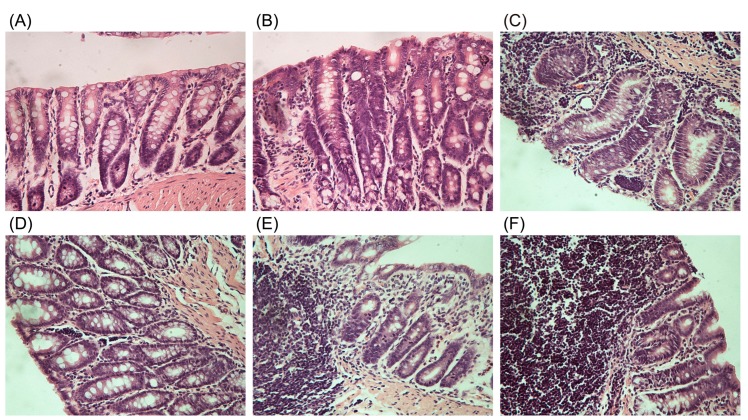
The H&E staining on the colons collected from the untreated rats and treated rats for over six days (original magnification 40×). (**A**) The colon from normal control rats (NC). (**B**) The colon from rats treated with the low dose of senna extract (L-SE). (**C**) The colon from rats treated with the high dose of senna extract (H-SE). (**D**) The colon from rats treated with the low dose of sennosides (L-SS). (**E**) The colon from rats treated with the high dose of sennosides (H-SS). (**F**) The colon from rats treated with the high dose of sennoside A (SA).

The paraffin embedded tissue sections from the six organs were performed with H&E staining and the images were captured under a microscope. No apparent pathological changes (data no shown) were observed in all the collected tissues excluding the colons (Figure 4). 

In the untreated rats (NC), the colons showed intact epithelium and mucosa, no disruption of crypt architecture, complete goblet cells with mucus-filled vacuoles, and no infiltration of leukocytes. 

However, moderate damage with decreased goblet cells were found in colons of the diarrhea-rats induced by a low dose of the senna products including the L-SE and L-SS. Severe lesions with decreased goblet cells were present in colons of the diarrhea-rats induced by the H-SE, H-SS, and SA. Particularly, inflammatory cell infiltration in the crypt architecture was found in diarrhea-rats induced by the SA treatment. 

### 2.5. Hypoxanthine Phosphoribosyl Transferase (Hprt) Is a Best Reference Gene for Aquaporin (Aqp) mRNA Expression Analysis in the Rats

**Figure 5:**
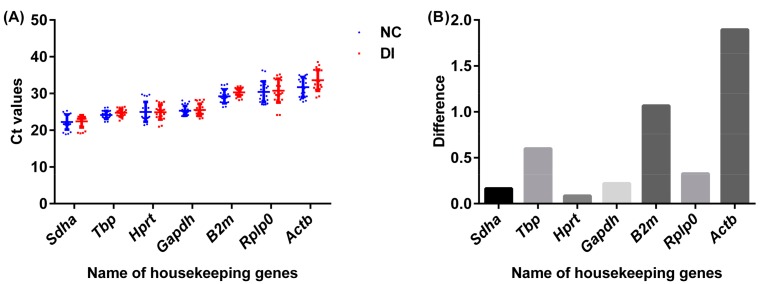
mRNA expression levels of seven housekeeping genes in normal and diarrhea-rat organs including colon, kidney, stomach, spleen, and liver. The expression was detected in 60 ng of RNA by using qRT-PCR. (**A**) Graphical representation of absolute *Cq* values for each gene analyzed in tissues collected from the normal rats (NC) and diarrhea-rats (DI) induced by senna extract. (**B**) The difference of mean *Cq* values for each gene between the normal rats and the diarrhea-rats. A line across the box depicts the median. Whiskers represent the maximum and minimum values.

To investigate the best reference gene for qRT-PCR analysis, seven housekeeping genes were investigated with expressions in the normal control rats and diarrhea-rats induced by the senna extract. The housekeeping genes selected in the study are glyceraldehyde-3-phosphate dehydrogenase (*Gapdh*), TATA box binding protein (*Tbp*), hypoxanthine phosphoribosyl transferase (*Hprt*), beta-2 micro-globulin (*B2m*), acidic ribosomal phosphoprotein (*Rplp0*), succinate dehydrogenase complex-subunit A (*Sdha*), and β-actin (*Actb*). The tissues used were colon, kidney, spleen, stomach, and liver. The *Cq* values of the seven housekeeping genes were detected and plotted in Figure 5A. 

Based on 60 ng of RNA, the *Cq* value shown in the normal group ranges from 22.25 to 31.72 and, in the diarrhea group, the range is between 22.41 and 33.62. They had a consistent result on overall ranking order, which was *Sdha* < *Tbp* < *Hprt* < *Gapdh* < *B2m* < *Rplp0* < *Actb*. *Sdha* had the highest expression with mean *Cq* values at 22.25 and 22.41 in normal and diarrhea rats, respectively. *Hprt* in the normal and diarrhea rats exhibited median *Cq* values with 25.00 and 24.91, respectively. The *Actb* in the normal and diarrhea rats had the highest *Cq* value with 31.72 and 33.62, respectively.

For expression stability analysis, the variation resulted from the treatment was investigated for each housekeeping gene (Figure 5B). The stability was determined based on the difference of mean *Cq* values between the diarrhea group and the normal group. The value of difference is: *Hprt* < *Sdha* < *Gapdh* < *Rplp0* < *Tbp* < *B2m* < *Actb*. *Actb* showed 5.98% variation and *B2m* had 3.65% with a statistic significant (*p* = 0.02). *Hprt* had a minimum variation (0.34%) with median *Cq* values and was, therefore, used as a reference gene for *Aqp* expression analysis in our study.

### 2.6. The Aqp Alteration Profiles in Diarrhea-Rats Induced by the SE, SS, and SA

**Figure 6:**
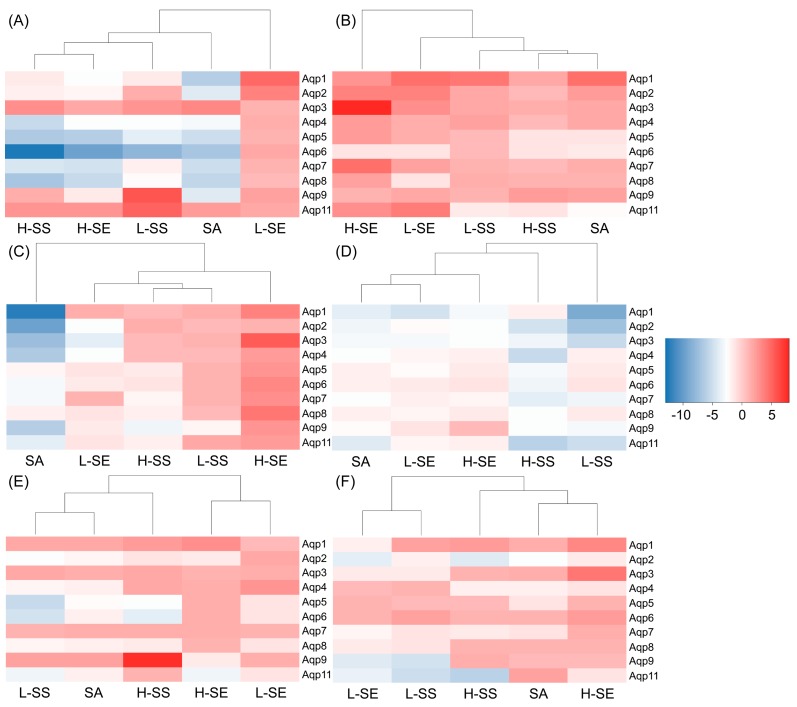
Heat map visualization with the *Aqp* alteration profiles in each organ collected from the diarrhea-rats induced by low dose of senna extract (L-SE), high dose of senna extract (L-SE), low dose of sennoside (L-SS), high dose of sennoside (H-SS), and one dose of sennoside A (SA) for over six days. (**A**) *Aqp* alteration profiles in the colons. (**B**) *Aqp* alteration profiles in the kidneys. (**C**) *Aqp* alteration profiles in the livers. (**D**) *Aqp* alteration profiles in the spleens. (**E**) *Aqp* alteration profiles in the stomachs. (**F**) *Aqp* alteration profiles in the lungs. The negative number represents the fold changes of down-regulated *Aqps* and the positive number represents the fold changes of up-regulated *Aqps*.

To look at the influence with the *Aqp* expressions on six organs in the rats, we used *Hprt* as a reference gene and quantified *Aqp* levels of ten subtypes including *Aqp1*, *Aqp2*, *Aqp3*, *Aqp4*, *Aqp5*, *Aqp6*, *Aqp7*, *Aqp8*, *Aqp9,* and *Aqp11*. The alteration profiles for each organ in the diarrhea groups were analyzed and figured as a heatmap (Figure 6). 

In the colons, similar patterns were found in all treated groups with the down-regulation on most of the *Aqps*. The dose-dependent relationship was found only in the SS groups with the expression on *Aqp4*, *Aqp5*, *Aqp6*, *Aqp7*, and *Aqp8*. Their expressions were down regulated by the H-SS to 12.57 times on *Aqp6*, to 6.96 times on *Aqp8*, to 6.59 times on *Aqp5*, to 5.47 times on *Aqp4*, and 4.54 times on *Aqp7*. *Aqp3*, however, was up regulated in all diarrhea groups and the maximum change is 3.47 times shown in group SA (Figure 6A). 

In the kidneys, there were minor alterations in the SA and SS groups, but there were major alterations in the SE groups. Up-regulations with most of the *Aqps* were found in the SE in both groups (Figure 6B). The dose-dependent manner was shown on *Aqp3* and *Aqp7* in group H-SE with the fold changes at 13.35 and 4.56 times, respectively.

In the livers, minor alterations were shown in group L-SS, H-SS, and L-SE without consistent patterns. The SS group on both had minor down regulation with the fold changes smaller than 1.90 times. However, *Aqp9* was exclusively down-regulated and dependent on the dose of SS with the fold changes at 2.00 and 3.15 times, respectively. The larger alterations were also found in group SA and H-SE but in opposite alteration profiles. In the group SA, down-regulation was shown on all of the *Aqps* in which *Aqp9* was regulated down to 6.38 times. In the group H-SE, up-regulation was shown on all of the *Aqps* with the fold changes from 1.25 to 5.55 times (Figure 6C). However, no dose-dependent manner was found in the SE groups.

In the spleens, minor down regulations on all of the *Aqps* were shown in all of the diarrhea groups (Figure 6D). Group H-SS was exclusive and had a greater influence with fold changes greater than 2.54 times. However, no dose-dependent manner was found in the SS groups. 

In the stomachs, minor alterations were shown on most of the *Aqps* in all the diarrhea groups (Figure 6E). No dose-dependent relationship was found in the *Aqps* excluding *Aqp9*. The up-regulation on *Aqp9* showed fold changes with 2.09 and 7.51 times relative to the L-SS and H-SS.

In the lungs, the *Aqp* changes were minor without similar dose-dependent patterns in all the diarrhea groups (Figure 6F).

Overall, in the colons, the down-regulation on the *Aqps* expressions was the characteristic profile for all the diarrhea rats in which the SS treated rats showed prominent changes with the down-regulation. The *Aqps* up-regulations shown in the kidneys and livers were greater in the SE treated rats than in the SS treated rats. The SA treatment had a distinguished profile on the livers with down-regulation on all of the *Aqps*. 

### 2.7. The Different Actions from the SE, SS, and SA Indicated by Principle Component Analysis (PCA) 

**Figure 7:**
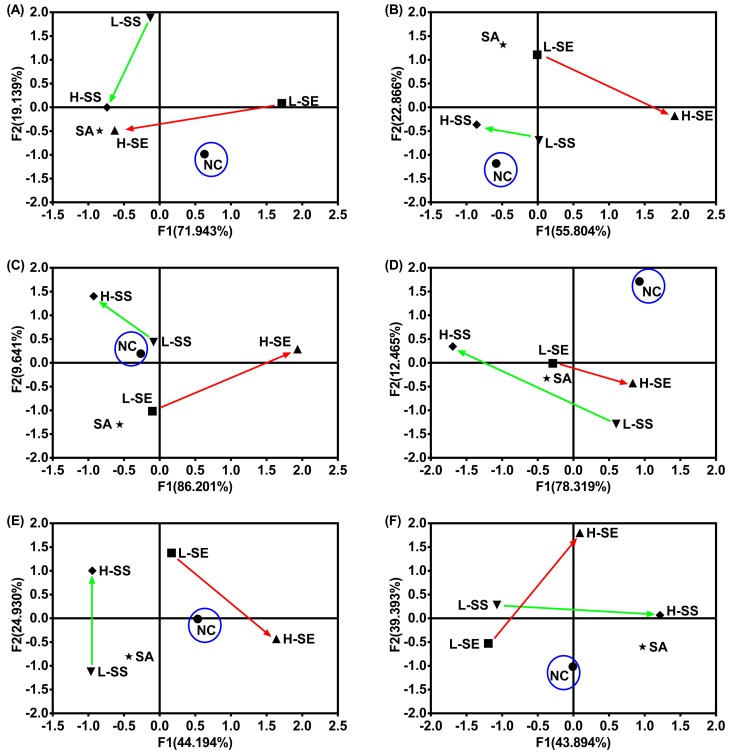
PCA results using the multiple *Aqps* expressions in organs collected from the normal rats and diarrhea-rats induced by the low dose of senna extract (L-SE), high dose of senna extract (L-SE), low dose of sennoside (L-SS), high dose of sennoside (H-SS), and one dose of sennoside A (SA) for over six days. (**A**) PCA results in the colons; (**B**) PCA results in the kidneys; (**C**) PCA results in the livers; (**D**) PCA results in the spleens; (**E**) PCA results in the stomachs; (**F**) PCA results in the lungs. The green arrow represents the trend from the L-SS to H-SS. The red arrow represents the trend from the L-SE to H-SE. The blue circle refers to the NC group.

To establish a reliable and integrative method to distinguish the difference among the treatments with each senna product, PCA was applied for species discrimination and integrative quality evaluation. The PCA results are illustrated in Figure 7, which displays four distinct clusters among the groups including the normal control (NC), low dose of SE (L-SE), high dose of SE (H-SE), low dose of SS (L-SS), high dose of SS (H-SS), and sennoside A (SA) (Figure 7). The same cluster represents similar *Aqps* alteration profiles displayed in the organ. The influence from the treatment was evaluated in terms of the distance between the treated group and the NC group. The farther distance implied the greater difference. 

In the colons, there were clearly differences between the normal and diarrhea groups (Figure 7A). The SS groups on both are localized in the same one cluster. The group H-SE and SA are in another cluster and the L-SS is in an individual cluster. The group NC is clearly separated in an individual cluster and is far away from the SS groups.

In the kidneys (Figure 7B) and livers (Figure 7C), similar patterns were observed with three clusters. The SS groups on both are localized in the same cluster with the group NC. The other two clusters are clearly distinguished by the group H-SE in one and groups L-SE and SA in another. 

In the spleens, the group NC is localized in an individual cluster and is far away from the other groups shown in three clusters (Figure 7D). The group L-SS and H-SE are in one cluster. L-SE and SA are in the other cluster. The group H-SS is in an individual cluster. Comparatively, the SS groups are far away from the NC group.

In the stomach, no big difference was observed among the groups (Figure 7E). The group NC is localized at the center and has a similar distance to the diarrhea groups. The group L-SS and SA are in the same cluster and the others are in three separated clusters.

In the lung, four clusters were fully occupied by the groups (Figure 7F). The group NC is localized at the vertical axis and shows a similar distance to the SE and SS groups. The group H-SE and H-SS are in the same cluster and others are in three separated clusters.

Overall, the SS groups are localized far away from the group NC in the colons and spleens. The SE groups are far away from the group NC in the livers and kidneys. No obvious difference with the distance to the group NC is shown in the stomachs and lungs.

## 3. Discussion

AQPs are known to facilitate transmembrane water fluxes in prokaryotes and eukaryotes. Thirteen AQP subtypes are differentially expressed in various organs and also have their own functions with conducting ions, glycerol, urea, CO_2_, nitric oxide, and other small solutes. Their alterations could result in the disturbance of molecule selection and water transport by AQPs leading to a pathological environment in intracellular and extracellular cells. For example, there is a relationship between the altered human AQP3 and AQP4 mRNA expression and gastritis types [11]. Upregulated AQP5 mRNA has been found in all of the 17 kidney biopsies from patients with diabetic nephropathy [12]. In addition, the up regulation on *Aqp3* mRNA were also found in constipation-rats induced by morphine [13]. Our results also showed that *Aqps* mRNA expressions were changed in the diarrhea-rats induced by the senna products. Therefore, looking at the AQP changes at transcriptional levels might be a useful method for insight into drug actions and toxicities on organs.

qRT-PCR is a sensitive and specific method for detecting changes in gene expression when an appropriate normalization strategy is established. Data normalization is required to avoid system errors introduced by multi-tissues and the multi-stages process including isolation and the RNA process [14]. To identify appropriate reference genes for *Aqps* detection, we investigated seven housekeeping genes that are expressed in various levels and are most commonly used in literature. As a reference gene, the mRNA levels should be stably expressed in various tissues and is not influenced by the drugs used. Surprisingly, most studies used *Gapdh* or *Actb* as a reference gene without proper validation protocols, which results in controversial outcomes. Our studies indicated that the expression of *Actb* was changed the most following the senna treatments in the rats. Thus, it would be very beneficial to use *Actb* as a reference gene. Our study demonstrated that *Hprt* is a best reference gene in rats for the AQPs expression assessment. 

AQPs in colon have been demonstrated with their important roles in many studies [11]. It is known that the colon epithelium absorbs about 1.5–2 L/day of water against an osmotic gradient with most moving mainly through AQPs [15]. Our studies showed that the treatment with the SE, SS, and SA could down regulate multiple *Aqp* expressions in the colons. The down regulation on *Aqps* could prevent water from being transmitted into the cells but could re-absorb in the colon, which leads to a high water content in rat feces. In the study, greater changes were found on multiple water-specific *Aqps* including *Aqp4*, *Aqp5*, *Aqp6*, *Aqp7*, and *Aqp8*. 

AQP4, which is predominantly located in the central nervous system, is permeable to water [16,17] and CO_2_ [18]. It is also expressed in the basolateral membrane of surface epithelial cells in the colon. A study suggested that AQP4 is involved in the rapid return of luminal water back into the body, which dehydrates the fecal contents [19]. In *Aqp4*-deficient mice, a high water content in feces and a reduced water osmotic permeability were observed [20]. The association between the down-regulated AQP4 and diarrhea has also been demonstrated in many diarrhea-related diseases. For example, the expression of AQP4 was significantly decreased in cholera toxin induced diarrhea [21], IBD patients [22], and allergic diarrhea [23]. 

AQP5 is expressed in glandular epithelia, alveolar epithelium, and secretory glands where it is involved in the generation of saliva, tears, and pulmonary secretions [24,25]. AQP5 is permeable to water and CO_2_ [18,26], but not much is known about the correlation between AQP5 dysfunction and disease [27]. The information with the distribution and localization in the colon are still unknown. 

AQP6 was first identified in the kidney of the rat, but the distribution and expression at both mRNA and protein levels was also demonstrated in the rat colon and the cecum [28]. It appears impermeable to H_2_O [18,29] and also enables transport of urea, glycerol, and nitrate [30,31].

AQP7 are mainly expressed in the apical parts of human colonic tissues with some cytoplasmic and basolateral distributions [22]. AQP7 immuno-reactivity was detected in the basolateral epithelia in the villi and the crypt of the human colon [32]. For rats, AQP7 is present on the apical region of the enterocytes in the villi, epithelial cells of the colon, and caecum, which suggests its involvement in rapid fluid movement through the villus epithelium [33]. AQP7 also facilitates the transport of water, glycerol, urea, ammonia, arsenite, and NH_3_ [18]. Abnormal regulation of glycerol is a remarkable contributing factor for the development of metabolic disease. 

AQP8 was first identified in intracellular domains of the proximal tubule and the collecting duct cells [34]. However, its transcript has been found to be widely expressed in the digestive system including the salivary glands, small intestine, colon, pancreas, and liver. In the human colon, AQP8 is expressed in the apical sides of the villus and crypt epithelial cells [32,35]. The inhibition of AQP8 expression by siRNA significantly decreased the osmotic water permeability in isolated superficial colonocytes in the rat proximal colon [36]. On the other hand, in 2,4,6-trinitrobenzene sulfonic acid (TNBS)-induced colitis model, which mimics human Crohn’s disease, AQP8 expression is downregulated with the increase of inflammation and injury [37], which indicates that AQP8 is possibly involved in inflammatory bowel disease. 

From above, we could presume that the senna metabolites might exert laxative function by targeting multiple *Aqps*. Their alterations might not only change water homeostasis but also modulate small solute transport in the colons. These changes could function with laxative effects but could be harmful/toxic to the colons. Under long-term use with senna products, the changes might not be impaired. In our study, the visible damage could be found in the colons of the rats administered with the senna products for over six days.

It is worth looking at the regulation on AQP3 in the colons. AQP3 is highly expressed in the esophagus and the proximal and distal colon [37]. In the human colon, AQP3 is expressed predominantly in mucosal epithelial cells [9]. It has been reported that an increase or decrease in AQP3 is involved in the onset of constipation or diarrhea [13,38]. Down-regulation of *Aqp3* was shown immediately in the colon of rats treated with senna products, but up-regulation was found in the rats with repeated administration of senna products for seven days [39]. Our consistent results showed that *Aqp3* expression was upregulated in the colon after the repeated administration with the three senna products for more than six days. Senna tolerate resulted from long-termed administration. We presume that the initial treatment with senna might result in a water dehydration status in mucosal epithelial cells. To facilitate water uptake, the cells might have active AQP3 through up-regulation, which might be one of the mechanisms for senna tolerate. 

The kidney and the liver are major organs of drug metabolites and, therefore, could be easily damaged. In this study, the up-regulation of AQP3 and AQP7 in the kidney was prominent in the diarrhea rats induced by the SE. AQP3 and AQP7, which are aquaglyceroporins, belong to transporters of water, glycerol, and H_2_O_2_. AQP3 is localized at the basolateral membranes of principal cells in kidney collecting duct. AQP7 is predominantly expressed on the apical membrane of the proximal straight tubules (S3 segment) [40,41]. AQP3 deletion in mice impaired urine concentrating ability and increased urine output [42]. AQP7 null mice do not show a urinary concentrating defect except the glycerol concentration in urine [43,44]. These studies indicated that AQP3 and AQP7 co-function to regulate water transport as well as to mediates the glycerol reabsorption in the proximal straight tubules. Although we could not find observed damage in kidney based on the immuno-histopathological examination, the potential influences from the SE could be revealed by the significant increased kidney index and distinguished *Aqp* expression profiles shown in the PCA analysis. 

The liver, which is another important organ, plays a central role in clearing drug metabolisms. The complex senna metabolites produced in intestines make it more challenging to evaluate the hepatotoxicity of senna products. Regarding the AQP regulations, we could concern the regulation of AQP9 in the liver following treatment with the senna products. AQP9 is highly predominantly expressed at the sinusoidal plasma membrane of hepatocytes [45] and function as a glycerol channel to facilitate glycerol uptake. It is permeable to water as well as glycerol, urea, carbamides, CO_2,_ and NH_3_. Deficiency of AQP9 could attenuate H_2_O_2_-induced cytotoxicity in human and mice cells, which indicates that AQP9-mediated H_2_O_2_ may regulate redox-regulated downstream cell signaling [46]. Our results showed that *Aqp9* expression was down regulated in the SA and SS groups but was up regulated in the SE groups. In addition, the SE-treated rats showed a significant increased liver index and distinguished *Aqp* expression profiles observed in the PCA analysis. This implied that the SE had more toxicity to the liver than the SS. Coincidentally, sennoside A, which are the main components of sennosides, was reported to protect mitochondrial function and structure to improve hepatic steatosis by inhibiting the mitochondrial respiratory chain complex I and the voltage-dependent anion channel 1 (VDAC1) [47]. We presume that the *Aqp9* up-regulated by the SS and SA might function to protect the liver against the influence/cytotoxicity induced by the SE. 

Up to date, it has been known that sennosides are the main bioactive components and have more powerful laxative functions than senna crude extracts. This effect was also demonstrated in our study with the fact that less SS was required to generate the diarrhea rats. It is also in line with our expectations that the overall alterations on *Aqps* expressions in the colons were more prominent in the diarrhea-rats induced by the SS. Thus, looking at the *Aqp* changes at transcriptional levels is a useful method for insight into actions from the product of senna.

In summary, we demonstrated that *Hprt* is the best reference gene for *Aqp* expression determination when using qRT-PCR. The analysis on *Aqps* alteration profiles could discriminate the actions from the SE, SS, and SA. We suggest that the SE might have higher potential influence/toxicity on the kidney and liver. The SS have a more powerful function on laxative effects but have less influence/toxicity on the kidney and liver. Thus, caution should be particularly exercised in the patients with kidney and liver diseases when chronic using senna-based products. Although these assays were performed in animals, the findings may lead to clinical applications from the administration amount to the selection of senna products for special patients.

## 4. Materials and Methods

### 4.1. Reagents and Materials 

All organic solvents applied for the extraction and separation were of an analytical grade and purchased from Tianjin Kemiou Chemical Reagent Co., Ltd., Tianjin, China. Acetonitrile used for HPLC analysis were of a chromatographic grade and was purchased from Merck (KGaA, Darmstadt, Germany). Ultrapure water was produced by a reverse osmosis Milli-Q (18 MΩ) system (Qingdao Flom Technology Co., Ltd., Qingdao, China). Senna leaves were purchased from commercial sources: (IMQ TCI Development Co., Ltd., Xi’an, China). Sennoside A (SA) for reference was obtained from Weikeqi Bio-Tech Co. Ltd. (Chengdu, China). 

### 4.2. Preparation and Analysis of Senna Products: SE, SS, and SA

Dried senna leaves were extracted with 50% ethanol and the solvent was evaporated in vacuum to obtain Senna extracts (SE). The SE dispersed in water was poured onto a D101 Macroporous adsorption resin column (Sunresin Inc., Xi’an, China), which was then eluted with water (till the eluent became colorless) followed by 60% ethanol (six-fold of column volume) successively. Sennosides (SS) were achieved in 60% ethanol eluent evaporated in vacuum to dryness.

The content of SA in the SE and SS were quantified by using the HPLC method recommended by Chinese Pharmacopoeia (2015) [48]. Chromatographic separations were achieved on an Hedera Si C18 column (4.6 × 250 mm, 5 μm, Hanbang Sci. & Tech, Huai’an, China) at 40 °C and the detection wavelength was 340 nm with a mobile phase consisting of acetonitrile-water (35:65) containing 5 mM tetraheptylmagnesium bromide (pH5.0) at a flow rate of 1 mL/min. The HPLC apparatus was a Waters Alliance 2695 (Waters, Milford, MA, USA) consisting of a quaternary pump, an autosampler, a thermostated column compartment, and a UV detector. All modules and data collection were controlled by Waters Empower2 software. 

### 4.3. Rats and Diarrhea Models Induced by the SE, SS, and SA 

Sixty male Sprague Dawley (SD) rats weighing 180–200 g were purchased from Dashuo experimental animal Co., Ltd. (Chengdu, China) (certificate No. SCXK-2014-028, 18 August 2014, Sichuan Provincial Laboratory Animal Public Service Center). Prior to the experiment, all animals were acclimatized in a pathogen-free-grade animal room under controlled conditions (24 ± 1.0 °C, 60 ± 5% humidity with a 12 h/12 h light-dark cycle) for three days and received standard laboratory chow and tap water *ad libitum*. All procedures for the care and handling of animals used in the study were approved by the Animal Care Committee of Northwest University (ACCNU-2016-0025). 

For the experiments, rats were randomly divided into six groups: normal control (NC), low dose of the SE (L-SE), high dose of the SE (H-SE), low dose of the SS (L-SS), and high dose of the SS (H-SS) and Sennoside A (SA) with eight rats in each group. The rats were intragastric administrated daily with 2.0 mL of drug solution or distilled water for each in six consecutive days. During the drug-dosing period, clinical signs were observed and recorded daily including body weight, stool consistency, and behavior status. They were given free access to food and water throughout the study. At day 6, rats were euthanized by intraperitoneal injection of ethyl carbamate (1 g/kg body weight) after 4 h of the last administration and were collected with six organs including stomach, spleen, liver, lung, kidney, and colon. The organs were weighted immediately, which was followed by the process further. Organs collected from two rats in each group were fixed in 4% paraformaldehyde and embedded in paraffin for hematoxylin and eosin (H&E) staining; from six rats were stored at −80 °C for mRNA extraction. 

### 4.4. Observation with Clinical Signs in Rats and Hematoxylin and Eosin (H&E) Staining on Tissue Slides 

The clinical signs were recorded with body weight, fecal water content, and behavior. Feces were observed by the water content and set as three grade (0, normal; +, loose stool; ++, watery diarrhea). Body weight was evaluated based on loss for each administration day. Behavior was observed especially regarding hair removal, diet reduction, and mental malaise. 

The tissues embedded in paraffin were sectioned into 0.45 μm slices that were placed on glass slides (colon, lung, stomach, spleen, kidney, and liver). The slides were deparaffinized and stained with hematoxylin and eosin. The slides were dehydrated in alcohol, cleared in exylene, and covered for imaging under a light microscope. The assessments were assigned for the destruction of the crypt structure, the depth of the lesions, and the degree of inflammatory cell infiltration.

### 4.5. Primer Design and Specificity

We designed primers for 17 genes which are *Sdha*, *Tbp*, *Hprt*, *Gapdh*, *B2m*, *Rplp0*, *Actb*, *Aqp1*, *Aqp2*, *Aqp3*, *Aqp4*, *Aqp5*, *Aqp6*, *Aqp7*, *Aqp8*, *Aqp9*, and *Aqp11*. The primers (Supplementary Table S1) used in the study were obtained by using a web-based tool (qPrimerDepot) [49]. The RT-qPCR products ranged from 100 to 300 bp. The specificities of all the primers were demonstrated by the single bands of an expected size in agarose gel electrophoresis and by the single-peak melting curves of the RT-qPCR products.

### 4.6. Extraction of Total RNA from Rat Tissues

Tissue samples were homogenized in TRIzol reagent (Sangon Biotech Co., Ltd., Shanghai, China) by using a tissue grinder (Ningbo Scientz Biotechnology Co., Ltd., Ningbo, China) under 4 °C. Total RNA was extracted by using the Trizol Reagent, according to the manufacturer’s instructions. The concentration of RNA was determined by using a NanoDrop 1000 Spectrophotometer (GE Co., Boston, MA, USA). The quality of RNA was checked using the 260/280 nm ratio with the values ranging from 1.8 to 2.0. All samples were stored at −80 °C until further analysis.

### 4.7. cDNA Synthesis and Real Time PCR

cDNA was synthesized from total RNA by a reverse transcriptase polymerase chain reaction (RT-PCR) using random hexamer primers and TaqMan Reverse Transcription Reagents (Sangon Biotech Co., Ltd., Shanghai, China). The cDNA (60 ng) was subjected to real-time PCR quantification after being mixed with a SYBR Green Real-Time PCR Master Mix (Sangon Biotech Co., Ltd., Shanghai, China). Various sets of gene-specific forward and reverse primers were listed in the Supplementary Table S1. All reactions were performed in triplicate in a final volume of 20 μL and analyzed with a Bio-Rad real-time PCR system (CFX Connect, Bio-Rad Laboratories Inc., Hercules, CA, USA). The thermal cycle used was 10 min at 95 °C, denaturing for 15 s at 95 °C, and annealing for 30 s at °C. PCR amplification was performed for 40 cycles. The *Cq* values greater than 35 was thought to be in non-temple controls (NTC). The data are expressed as expressions relative to that of *Hprt* using the 2^−∆∆*C*t^ method [50]. The mRNA levels are the mean of values obtained from at least three rats in each group.

### 4.8. Heatmap and PCA

To demonstrate overall alteration profiles for each diarrhea group, the visualization form of the heat map and PCA were conducted by the heat map function in the R program (version 3.5.1, Free Software Foundation, Inc., Boston, MA, USA) and SPSS 19.0 software (GraphPad Software, Inc., La Jolla, CA, USA), respectively.

### 4.9. Statistical Analysis

Statistical analyses were performed by using SPSS 19.0 software and GraphPad Prism version 6.0 (GraphPad Software, Inc., La Jolla, CA, USA). All results are expressed as means ± SD. The parametric test was used in the statistical analysis. The normality of the data was verified by using the Kolmogorov-Smirnov test. Then the comparisons between the groups were made by one-way ANOVA and were followed by Dunnett’s test for multiple comparisons. Results with *p* < 0.05 was considered to be statistically significant.

## Figures and Tables

**Table 1:** Diarrhea-rats generation by feeding the senna products.

Group	Senna Products	Feeding Amount	Diarrhea Grade	Content of SA
L-SE	Low dose of SE	1725.00 mg/kg	+	20.65 mg/kg
H-SE	High dose of SE	3450.00 mg/kg	++	41.30 mg/kg
L-SS	Low dose of SS	156.94 mg/kg	+	12.76 mg/kg
H-SS	High dose of SS	313.88 mg/kg	++	25.52 mg/kg
SA	sennoside A	40.00 mg/kg	+	50.00 mg/kg

Senna Extract (SE), Sennosides (SS), and Sennoside A (SA).

## Supplementary Materials

The following are available online at http://www.mdpi.com/1422-0067/19/10/3210/s1.

## References

[1] Sun S.W., Su H.T. (2002). Validated HPLC method for determination of sennosides A and B in senna tablets. J. Pharm. Biomed. Anal..

[2] Van Gorkom B.A.P., de Vries E.G.E., Karrenbeld A., Kleibeuker J.H. (1999). Anthranoid laxatives and their potential carcinogenic effects. Aliment. Pharmacol. Ther..

[3] Vilanovasanchez A., Gasior A.C., Toocheck N., Weaver L., Wood R.J., Reck C.A., Wagner A., Hoover E., Gagnon R., Jaggers J. (2018). Are Senna based laxatives safe when used as long term treatment for constipation in children?. J. Pediatr. Surg..

[4] Sakulpanich A., Gritsanapan W. (2009). Laxative anthraquinone contents in fresh and cooked *Senna siamea* leaves. Southeast Asian J. Trop. Med. Public Health.

[5] Agre P. (2006). The Aquaporin Water Channels. Proc. Am. Thorac. Soc..

[6] Benga G. (2012). On the definition, nomenclature and classification of water channel proteins (aquaporins and relatives). Mol. Aspects Med..

[7] Ishibashi K., Hara S., Kondo S. (1997). Aquaporin water channels in mammals. Clin. Exp. Nephrol..

[8] Laforenza U. (2012). Water channel proteins in the gastrointestinal tract. Mol. Aspects Med..

[9] Ikarashi N., Kon R., Sugiyama K. (2016). Aquaporins in the Colon as a New Therapeutic Target in Diarrhea and Constipation. Int. J. Mol. Sci..

[10] Zhang Y., Wang X., Sha S., Liang S., Zhao L., Liu L., Chai N., Wang H., Wu K. (2012). Berberine increases the expression of NHE3 and AQP4 in sennosideA-induced diarrhoea model. Fitoterapia.

[11] Cui Z., Zhuang C., Jiang Z. (2016). Expression, distribution and role of aquaporin water channels in human and animal stomach and intestines. Int. J. Mol. Sci..

[12] Li Y., Wang W., Jiang T., Yang B. (2017). Aquaporins in Urinary System. Adv. Exp. Med. Biol..

[13] Kon R., Ikarashi N., Hayakawa A., Haga Y., Fueki A., Kusunoki Y., Tajima M., Ochiai W., Machida Y., Sugiyama K. (2015). Morphine-Induced Constipation Develops with Increased Aquaporin-3 Expression in the Colon via Increased Serotonin Secretion. Toxicol. Sci..

[14] Huggett J., Dheda K., Bustin S., Zumla A. (2005). Real-time RT-PCR normalisation; strategies and considerations. Genes Immun..

[15] Soyuncu S., Cete Y., Nokay A.E. (2008). Portal vein thrombosis related to *Cassia angustifolia*. Clin. Toxicol..

[16] Yang B., Brown D., Verkman A.S. (1996). The Mercurial Insensitive Water Channel (AQP-4) Forms Orthogonal Arrays in Stably Transfected Chinese Hamster Ovary Cells. J. Biol. Chem..

[17] Yang B., Ma T., Verkman A.S. (1995). cDNA cloning, gene organization, and chromosomal localization of a human mercurial insensitive water channel. Evidence for distinct transcriptional units. J. Biol. Chem..

[18] Geyer R.R., Musa-Aziz R., Qin X., Boron W.F. (2013). Relative CO_2_/NH_3_ selectivities of mammalian aquaporins 0-9. Am. J. Physiol. Cell Physiol..

[19] Guttman J.A., Samji F.N., Li Y., Deng W., Lin A., Finlay B.B. (2010). Aquaporins contribute to diarrhoea caused by attaching and effacing bacterial pathogens. Cell. Microbiol..

[20] Wang K.S., Ma T., Filiz F., Verkman A.S., Bastidas J.A. (2000). Colon water transport in transgenic mice lacking aquaporin-4 water channels. Am. J. Physiol. Gastrointest. Liver Physiol..

[21] Hamabata T., Liu C., Takeda Y. (2002). Positive and negative regulation of water channel aquaporins in human small intestine by cholera toxin. Microb. Pathog..

[22] Hardin J.A., Wallace L.E., Wong J.F., O’Loughlin E.V., Urbanski S.J., Gall D.G., Macnaughton W.K., Beck P.L. (2004). Aquaporin expression is downregulated in a murine model of colitis and in patients with ulcerative colitis, Crohn’s disease and infectious colitis. Cell Tissue Res..

[23] Yamamoto T., Kuramoto H., Kadowaki M. (2007). Downregulation in aquaporin 4 and aquaporin 8 expression of the colon associated with the induction of allergic diarrhea in a mouse model of food allergy. Life Sci..

[24] Song Y., Sonawane N., Verkman A.S. (2010). Localization of aquaporin-5 in sweat glands and functional analysis using knockout mice. J. Physiol..

[25] Song Y., Verkman A.S. (2001). Aquaporin-5 dependent fluid secretion in airway submucosal glands. J. Biol. Chem..

[26] Musa-Aziz R., Chen L.M., Pelletier M.F., Boron W.F. (2009). Relative CO_2_/NH_3_ selectivities of AQP1, AQP4, AQP5, AmtB, and RhAG. Proc. Natl. Acad. Sci. USA.

[27] Direito I., Madeira A., Brito M.A., Soveral G. (2016). Aquaporin-5: From structure to function and dysfunction in cancer. Cell Mol. Life Sci..

[28] Laforenza U., Gastaldi G., Polimeni M., Tritto S., Tosco M., Ventura U., Scaffino M.F., Yasui A.M. (2009). Aquaporin-6 is expressed along the rat gastrointestinal tract and upregulated by feeding in the small intestine. BMC Physiol..

[29] Liu K., Kozono D., Kato Y., Agre P., Hazama A., Yasui M. (2005). Conversion of aquaporin 6 from an anion channel to a water-selective channel by a single amino acid substitution. Proc. Natl. Acad. Sci. USA.

[30] Holm L.M., Dan A.K., Zeuthen T. (2004). Aquaporin 6 is permeable to glycerol and urea. Pflugers Arch..

[31] Ikeda M., Beitz E., Kozono D., Guggino W.B., Agre P., Yasui M. (2002). Characterization of aquaporin-6 as a nitrate channel in mammalian cells. Requirement of pore-lining residue threonine 63. J. Biol. Chem..

[32] Ricanek P., Lunde L.K., Frye S.A., Støen M., Nygård S., Morth J.P., Rydning A., Vatn M.H., Amirymoghaddam M., Tønjum T. (2015). Reduced expression of aquaporins in human intestinal mucosa in early stage inflammatory bowel disease. Clin. Exp. Gastroenterol..

[33] Laforenza U., Gastaldi G., Grazioli M., Cova E., Tritto S., Faelli A., Calamita G., Ventura U. (2005). Expression and immunolocalization of aquaporin-7 in rat gastrointestinal tract. Biol. Cell.

[34] Elkjaer M.L., Nejsum L.N., Gresz V., Kwon T.H., Jensen U.B., Frøkiaer J., Nielsen S. (2001). Immunolocalization of aquaporin-8 in rat kidney, gastrointestinal tract, testis, and airways. Am. J. Physiol. Renal Physiol..

[35] Fischer H., Stenling R., Rubio C., Lindblom A. (2001). Differential expression of aquaporin 8 in human colonic epithelial cells and colorectal tumors. BMC Physiol..

[36] Laforenza U., Cova E., Gastaldi G., Tritto S., Grazioli M., Larusso N.F., Splinter P.L., D’Adamo P., Tosco M., Ventura U. (2005). Aquaporin-8 Is Involved in Water Transport in Isolated Superficial Colonocytes from Rat Proximal Colon. J. Nutr..

[37] Zhao G., Li J., Wang J., Shen X., Sun J. (2014). Aquaporin 3 and 8 are down-regulated in TNBS-induced rat colitis. Biochem. Biophys. Res. Commun..

[38] Ikarashi N., Baba K., Ushiki T., Kon R., Mimura A., Toda T., Ishii M., Ochiai W., Sugiyama K. (2011). The laxative effect of bisacodyl is attributable to decreased aquaporin-3 expression in the colon induced by increased PGE2 secretion from macrophages. Am. J. Physiol. Gastrointest. Liver Physiol..

[39] Kon R., Yamamura M., Matsunaga Y., Kimura H., Minami M., Kato S., Ikarashi N., Sugiyama K. (2018). Laxative effect of repeated Daiokanzoto is attributable to decrease in aquaporin-3 expression in the colon. J. Nat. Med..

[40] Nejsum L.N., Elkjær M.L., Hager H., Frøkiær J., Kwon T.H., Nielsen S. (2000). Localization of Aquaporin-7 in Rat and Mouse Kidney Using RT-PCR, Immunoblotting, and Immunocytochemistry. Biochem. Biophys. Res. Commun..

[41] Ishibashi K., Imai M., Sasaki S. (2000). Cellular localization of aquaporin 7 in the rat kidney. Exp. Nephrol..

[42] Lei L., Wang W., Jia Y., Su L., Zhou H., Verkman A.S., Yang B. (2017). Aquaporin-3 deletion in mice results in renal collecting duct abnormalities and worsens ischemia-reperfusion injury. Biochim. Biophys. Acta.

[43] Sohara E., Rai T., Sasaki S., Uchida S. (2006). Physiological roles of AQP7 in the kidney: Lessons from AQP7 knockout mice. Biochim. Biophys. Acta.

[44] Maeda N., Funahashi T., Hibuse T., Nagasawa A., Kishida K., Kuriyama H., Nakamura T., Kihara S., Shimomura I., Matsuzawa Y. (2004). Adaptation to fasting by glycerol transport through aquaporin 7 in adipose tissue. Proc. Natl. Acad. Sci. USA.

[45] Elkjaer M., Vajda Z., Nejsum L.N., Kwon T., Jensen U.B., Amirymoghaddam M., Frøkiaer J., Nielsen S. (2000). Immunolocalization of AQP9 in liver, epididymis, testis, spleen, and brain. Biochem. Biophys. Res. Commun..

[46] Watanabe S., Moniaga C.S., Nielsen S., Hara-Chikuma M. (2016). Aquaporin-9 facilitates membrane transport of hydrogen peroxide in mammalian cells. Biochem. Biophys. Res. Commun..

[47] Le J., Jia W., Sun Y. (2018). Sennoside A protects mitochondrial structure and function to improve high-fat diet-induced hepatic steatosis by targeting VDAC1. Biochem. Biophys. Res. Commun..

[48] State Pharmacopoeia Commission (2015). Pharmacopoeia of the People’s Republic of China.

[49] Cui W., Taub D.D., Gardner K. (2007). qPrimerDepot: A primer database for quantitative real time PCR. Nucleic Acids Res..

[50] Livak K.J., Schmittgen T.D. (2001). Analysis of relative gene expression data using real-time quantitative PCR and the 2^−ΔΔ*C*t^ Method. Methods.

