# Supplementary material for: Aquaporins Alteration Profiles Revealed Different Actions of Senna, Sennosides, and Sennoside A in Diarrhea-Rats
Source: Int J Mol Sci. 2018 Oct 17;19(10):3210. doi: 10.3390/ijms19103210 (PMC6213963; doi:10.3390/ijms19103210)
Supplement: Supplementary file 1 [file ijms-19-03210-s001.pdf]

Supplementary Table S1: Primers used for qRT-PCR

Gene name	RefSeq ID	Primer sequence（5'-3'）	Amplicon size
Aqp1	NM_007472	F-TGCAGAGTGCCAATGATCTC<br>R-GGCATCACCTCCTCCCTAGT	104
Aqp2	NM_009699	F-GGAAGAGCTCCACAGTCACC<br>R-CCTCCATGAGATTACCCCTG	101
Aqp3	NM_016689	F-AAGCCAAGTTGATGGTGAGG<br>R-GGGGACCCTCATCCTTGT	98
Aqp4	NM_009700	F-TATCCAGTGGTTTGCCCAGT<br>R-GCAATTGGACATTTGTTTGC	105
Aqp5	NM_009701	F-TAGAAGATGGCTCGGAGCAG<br>R-CTGGGACCTGTGAGTGGTG	92
Aqp6	NM_175087	F-CAAAGACACAGAGCACCAGC<br>R-GAGAGACCCTTGGGGTCAAC	130
Aqp7	NM_007473	F-TGACACCGAGATAGCTGCC<br>R-GCGAGAGTTTCTGGCAGAGT	110
Aqp8	NM_007474	F-CACAGCAGGGTTGAAGTGTC<br>R-AGTCCGAATACTGGGCTCCT	108
Aqp9	NM_022026	F-TAGTGATGATCCCACCAGCC<br>R-CACTCTCTGAGTTCCTGGGC	102
Aqp11	NM_175105	F-AAGAGTTCGTCAAAGCACGG<br>R-TGCACTCATCACCTTTTTGG	105
Sdha	NM_023281	F-AACAGAGAAGTGAAAGCCGC<br>R-CGCAGTTTCGAGGCTTCTT	97
Tbp	NM_013684	F-GTGAAGGGTACAAGGGGGTG<br>R-ACATCTCAGCAACCCACACA	109
Hprt	NM_013556	F-CATAACCTGGTTCATCATCGC<br>R-TCCTCCTCAGACCGCTTTT	95
Gapdh	NM_001001303	F-TTGATGGCAACAATCTCCAC<br>R-CGTCCCGTAGACAAAATGGT	110
B2m	NM_009735	F-TTCAGTATGTTCGGCTTCCC<br>R-TGGTGCTTGTCTCACTGACC	103
Rplp0	NM_023281	F-CCGATCTGCAGACACACACT<br>R-ACCCTGAAGTGCTCGACATC	91
Actb	NM_007393	F-ATGGAGGGGAATACAGCCC<br>R-TTCTTTGCAGCTCCTTCGTT	149
